# Physical function varies according to characteristics of executive dysfunction in patients with stroke

**DOI:** 10.1016/j.ijchp.2026.100725

**Published:** 2026-09-26

**Authors:** Katsuya Sakai, Yuichiro Hosoi, Yusuke Harada, Yuichi Kato, Junpei Tanabe, Takayuki Miyauchi

**Affiliations:** a Department of Physical Therapy, Graduate School of Human Health Sciences, Tokyo Metropolitan University, Tokyo, Japan; b Nuffield Department of Clinical Neurosciences, University of Oxford, Oxford, United Kingdom; c Department of Rehabilitation Medicine, Keio University School of Medicine, Tokyo, Japan; d Department of Rehabilitation, Faculty of Health, Medical and Sports, Teikyo Heisei University, Chiba, Japan; e Department of rehabilitation, Reiwa Rehabilitation Hospital, Chiba, Japan; f Department of Rehabilitation, Moriyama Neurological Center Hospital, Tokyo, Japan; g Department of Physical Therapy, Hiroshima Cosmopolitan University, Hiroshima, Japan; h Department of Rehabilitation, Faculty of Medical Sciences, Shonan University of Medical Sciences, Kanagawa, Japan

**Keywords:** Stroke, Distribution pattern, Executive function, Physical function

## Abstract

**Introduction:**

While executive dysfunction significantly impacts physical function, most clinical assessments rely on total composite scores, potentially overlooking the heterogeneity of specific subcomponent impairments and their unique contributions to motor recovery. This study aimed to identify distinct profiles of executive dysfunction based on the score distribution of Frontal Assessment Battery (FAB) subitems and clarify their specific associations with motor and balance impairments in patients with stroke.

**Methods:**

Seventy-eight patients with subacute stroke were participated in this observational study. Assessments were used the FAB, Brunnstrom Recovery Stage (BRS), Berg Balance Scale (BBS), and Timed Up and Go Test (TUGT). Representational Similarity Analysis (RSA) and k-means clustering were applied to FAB subitem scores to identify patient subgroups based on the similarity of their executive profiles.

**Results:**

Three distinct clusters were identified: Cluster 1 (Inhibitory Control Impairment, N = 32), Cluster 2 (Motor Programming Impairment, N = 18), and Cluster 3 (Mild, N = 28). Notably, no significant differences were observed among the clusters in total FAB scores or other global cognitive measures (P > 0.05). However, physical function significantly varied; Cluster 3 demonstrated significantly superior performance in BRS, BBS, and TUGT scores compared to Clusters 1 and 2 (P < 0.05), which exhibited lower physical function despite their different executive profiles.

**Conclusions:**

This study suggested that the individual differences in executive dysfunction—which cannot be observed in total scores—and motor dysfunction within clusters are associated with different factors.

## Introduction

Cognitive function encompasses multiple domains, including memory, attention, visuospatial, working memory, and executive function (Divandari et al., 2023). Among these, executive function represents a higher-order integrative system that regulates goal-directed behavior, incorporating processes such as planning, decision-making, inhibitory control, and cognitive flexibility (Skidmore et al., 2023). Contemporary models conceptualize executive function as a multidimensional construct comprising partially independent components—specifically inhibition, working memory, and cognitive flexibility—each contributing uniquely to behavioral outcomes (Miyake, & Friedman, 2012; Friedman & Miyake, 2017).

Executive dysfunction is highly prevalent following stroke, affecting 51.4% of patients (Sakai et al., 2024), and is closely associated with reduced independence in activities of daily living (ADL) (Sakai et al., 2024; Lipskaya-Velikovsky et al., 2018; Ownsworth, & Shum, 2008). Importantly, impairments in specific executive components, such as inhibitory control and cognitive flexibility, have been shown to distinctly influence functional outcomes, including attention and ADL performance (Sakai et al., 2025). These findings highlight the clinical importance of assessing executive function at the component level rather than relying solely on composite scores.

Previous studies have demonstrated significant associations between executive function and physical outcomes after stroke (Sakai et al., 2024; Sakai et al., 2023; Hayes et al., 2016; Hayes et al., 2013). For example, executive dysfunction has been linked to impairments in balance, gait, and motor recovery. Assessments using tools such as the Behavioral Assessment of the Dysexecutive Syndrome (BADS) have shown that patients with more severe executive deficits exhibit poorer postural control and reduced dynamic balance (Hayes et al., 2016). Furthermore, executive function has been suggested to influence physical performance indirectly, mediated by the severity of motor paralysis and functional independence (Sakai et al., 2024). In addition, executive dysfunction is associated with both single-task and dual-task walking abilities; specifically, patients with executive dysfunction exhibit reduced gait speed under both conditions (Sakai et al., 2024; Sakai et al., 2023; Hayes et al., 2011; Hayes et al., 2013).

Despite these findings, most studies have evaluated executive function using total scores (Hayes et al., 2013; Han et al., 2020; Sakai et al., 2024; Sakai et al., 2024), potentially overlooking the heterogeneity in the distribution of subcomponent impairments. Given its multi-component nature, patients with similar total scores may exhibit distinct profiles of executive dysfunction, reflecting different underlying mechanisms.

The Frontal Assessment Battery (FAB) is a concise clinical tool consisting of six subtests that assess different aspects of executive function, enabling fractionated evaluation (Sakai et al., 2024; Dubois et al., 2000). Previous principal component analyses have suggested that the FAB captures multiple underlying dimensions (Sakai et al., 2025; Wang et al., 2016), supporting its utility in identifying heterogeneous executive profiles. Therefore, this study aimed to examine whether profiles of executive dysfunction differ according to the distribution of FAB subitem scores and to clarify their specific associations with motor and balance impairments in patients with stroke.

## Methods

This observational study recruited patients at three rehabilitation hospitals from August 2022 to December 2025. There were no changes to the protocol during this period. The inclusion criteria were: (1) first-time stroke, (2) diagnosis of cerebral haemorrhage or infarction, (3) age >18 years. The exclusion criteria were as follows: (1) diagnosis of severe dementia and Alzheimer’s disease, (2) diagnosis of higher brain dysfunction (e.g. unilateral spatial neglect, aphasia, and apraxia), and (3) age >90 years. The sample size for the cluster analysis was determined based on a rule of thumb of N > 2p, where p represents the number of variables included in the cluster analysis (Ward, 1963; Sakai et al., 2026). Since the FAB included six subcategories, a sample size of at least 13 patients was required for each cluster. Patients received an explanation of the study’s purpose, after which written informed consent was obtained prior to study initiation. This study was approved by the ethics committee of the Ishikawajima Memorial Hospital, Reiwa Rehabilitation Hospital, and Moriyama Neurological Center Hospital (approval number: 2022-02, 00-10, and 23002, respectively). It is publicly registered in the UMIN Clinical Trials Registry (UMIN-CTR) (trial registration ID: UMIN000048587) and complies with the ethical standards established in the 1964 Declaration of Helsinki.

## Outcomes

All assessments were measured within five days. Cognitive assessments included the FAB, Mini-Mental State Examination (MMSE), Trail Making Test (TMT) Part A and B, and Functional Independence Measure (FIM) cognitive part. Motor function assessments included the Brunnstrom Recovery Stage (BRS), sensory part of stroke impairment assessment set (SIAS), Timed Up and Go Test (TUGT), Berg Balance Scale (BBS), and FIM motor part.

### Cognitive function

The FAB comprises six subscales, each scored from 0 to 3 points: item 1, similarities, assesses conceptualization; item 2, lexical-verbal fluency, assesses mental flexibility; item 3, motor series, assesses programming; item 4, conflicting instructions, assesses sensitivity to interference; item 5, go/no-go, assesses inhibitory control; and item 6, prehension behaviour, assesses environmental autonomy. The maximum total score is 18 points, with higher scores indicating better frontal lobe and executive functions (Sakai et al., 2024; Dubois et al., 2000).

The MMSE was used to assess cognitive function, with a total score of 30 points indicating good cognitive function (Perneczky et al, 2006).

TMT part A reflects motor speed and attention function, whereas part B is often used to assess executive functions (Tamez et al, 2011). For TMT part A, the patients connected circled numbers (from 1 to 25) in sequence as quickly as possible. In TMT part B, patients connected numbers and letters alternatingly in sequence as quickly as possible.

The FIM assessments measure the ability to perform ADL and comprise the FIM motor items (13 items) and FIM cognitive items (five items) (Granger, 1986). FIM cognitive items comprise communication (expression and comprehension) and social cognition (social interaction, problem solving, and memory), and they can be used to assess the cognitive functions necessary for daily life. Each item is rated on a six-point scale (score range, 1–7), with 35 points for the FIM cognitive items. Higher scores indicate better ADL cognitive function.

### Motor function

The BRS assesses the degree of motor paralysis of the upper limb, hand, and lower limb functions, and consists of six level. The BRS had higher scores on this index (score range, 1–6), indicating better motor function (Brunnstrom, 1966).

The SIAS is a comprehensive tool for assessing motor and sensory function in patients with stroke (Liu et al, 2002). In this study, only sensory items (tactile and position sensory) were used. This test was scored on a 4-point scale (score range: 0–3). The better the sensory function, the higher the score.

TUGT is an assessment tool for the dynamic balance and walking functions (Hafsteinsdottir et al., 2014). The patient was seated in a 40-cm chair. The patient was instructed to stand up from the chair, walk to a cone 3 m away from the chair, walk around it toward their nonparalytic side, return to the chair, and sit down (Sakai et al., 2024; Sakai et al., 2023). The assessor instructed the patient to stand up from the chair at the ‘ready to go’ cue and measured the time with a stopwatch. Patients were allowed to use walking aids and orthotics that they normally use in their daily life. The TUGT was performed twice at a maximum speed. The average TUGT was then calculated.

The BBS can measure the ability to maintain balance function (Berg et al., 1995). It comprises 14 items, and each item is scored on a 5-point scale (0–4), with a total score of 56 points. The BBS consists of basic movements such as standing up, one-leg standing, and turning. Higher scores indicate better balance function.

FIM motor item is rated on a six-point scale (score range, 1–7), with 91 points. Higher scores indicate better ADL motor function (Granger, 1986).

## Statistical analysis

### Representational Similarity Analysis and cluster analysis

Representational Similarity Analysis (RSA) was conducted using the six-item scores of FAB for each participant. Pairwise dissimilarities between patients were calculated using correlation distance, and a representational dissimilarity matrix (RDM) was constructed (Nili et al., 2014; Pacheco-Estefan et al., 2024). Multidimensional scaling (MDS) was applied to the RDM to obtain a two-dimensional representation. K-means clustering (k = 3) was then performed on the two-dimensional coordinates (Fig. 1).

**Fig. 1:**
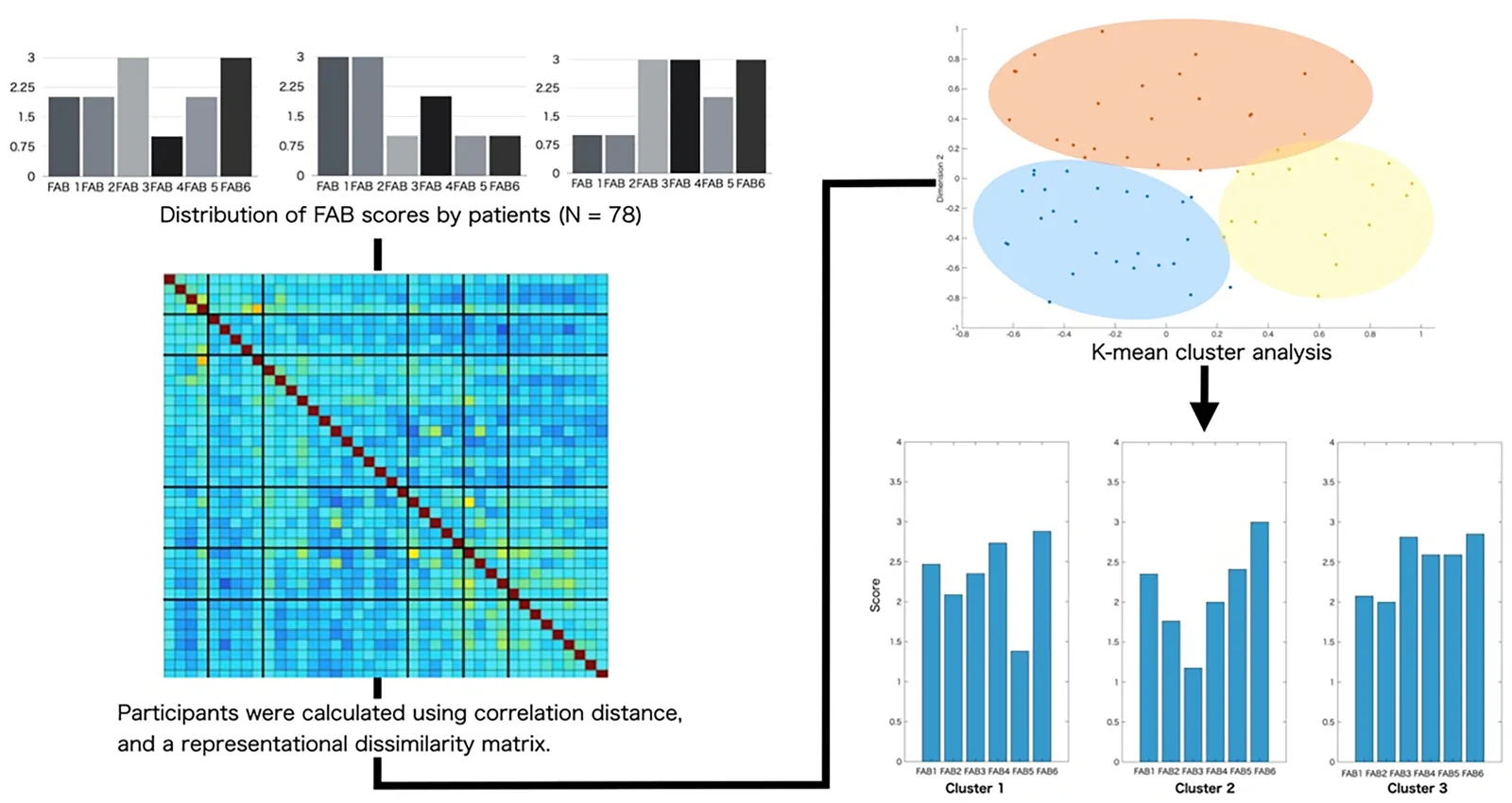
Analyses methods. The analysis involved creating a score distribution for 78 patients, followed by Representational Similarity Analysis. Since the Frontal Assessment Battery consists of six items and thus has six dimensions, we performed Multidimensional scaling analysis to reduce it to two dimensions and then classified the two-dimensional data using k-means clustering.

To determine the appropriate number of clusters, k-means clustering solutions with 2 to 6 clusters were evaluated using the mean silhouette coefficient. For each number of clusters, k-means clustering was performed with 100 random initializations, and the solution with the lowest within-cluster sum of squared distances was selected. The cluster solution with the highest mean silhouette coefficient was selected for subsequent analyses. The silhouette coefficient ranges from 1 to -1; the closer it is to 1, the more appropriate the number of clusters is (Kodinariya, & Makwana, 2013; Yuan, & Yang, 2019). Moreover, to assess the stability of the selected clustering solution against sampling variation, a nonparametric bootstrap procedure with 1,000 resamples was performed. For each bootstrap sample, the complete analytical procedure, including correlation-based distance calculation, two-dimensional MDS, and k-means clustering, was repeated using the same number of clusters as in the original analysis. The agreement between the original and bootstrap cluster assignments for the resampled participants was quantified using the adjusted Rand index (ARI). Because MDS configurations can differ in orientation due to arbitrary rotation or reflection, stability was assessed based on participant-level cluster assignments rather than direct comparison of MDS coordinates. The mean, median, standard deviation, and 95% percentile interval of the bootstrap ARI distribution were calculated.

### Linear mixed models and ordinal logistic regression

For the comparison between clusters, the statistical model was selected based on the scale of the dependent variable. Age was selected a priori as a covariate because of its potential influence on both cognitive and physical performance after stroke. For comparisons among the three clusters, continuous variables (FAB total, MMSE, TMT part A and B, TMT part B – A, TUGT, BBS, and FIM score) were analyzed using linear mixed models with age as a covariate. The overall cluster effect was quantified using partial eta squared (ηp²). For significant pairwise comparisons, Bonferroni-adjusted estimated marginal mean differences and their 95% confidence intervals were reported. Ordinal variables (FAB item 1 – 6, BRS, and SIAS) were analyzed using ordinal logistic regression with age as a covariate. Odds ratios (ORs) and corresponding 95% confidence intervals (CI) were reported to quantify the magnitude of associations between cluster membership and ordinal variables.

### Assessment of model assumptions and Sensitivity analyses

The distributional assumptions of the linear mixed-effects models were assessed using graphical diagnostic procedures. Quantile–quantile (Q–Q) plots of the model residuals were visually inspected to assess the normality of the residuals, and residual-versus-fitted value plots were examined to assess the homogeneity of variance and the presence of potentially influential observations. The Shapiro–Wilk test was additionally used as a quantitative assessment of residual normality, and the Breusch–Pagan test was used to assess heteroscedasticity.

Sensitivity analyses were performed for continuous outcomes that showed substantial deviations from the model assumptions in the residual diagnostic analyses. The same fixed-effects structure as in the primary analysis, including cluster as the main factor and age as a covariate, was retained for all sensitivity analyses. In addition, a robust sensitivity analysis using heteroscedasticity-consistent (HC3) standard errors was performed for TMT part B, TMT B−A, and TUGT. The results of the sensitivity analyses were compared with those of the primary models with respect to the overall cluster effect and the direction of the pairwise cluster differences.

RSA analysis was performed using MATLAB 2025a (MathWorks, Natick, MA, USA) and statistical analysis was performed using SPSS (version 29.0; SPSS Inc., Chicago, IL, USA), and the statistical significance was set at p < 0.05.

## Results

This study enrolled 104 inpatients with subacute stroke in three rehabilitation hospitals. According to the inclusion criteria and exclusion criteria, a total of 26 patients with stoke were excluded because of age (n = 2), not possible TUGT (n = 9), not possible TMT (n = 11), not possible FAB (n = 1), ataxia (n = 2), and missing data (n = 1). Finally, a total of 78 patients ultimately participated in the study (mean age: 67.8 ± 12.6 years; sex: male 46, female 32; mean time since stroke: 54.1 ± 53.3 days, Fig. 2).

**Fig. 2:**
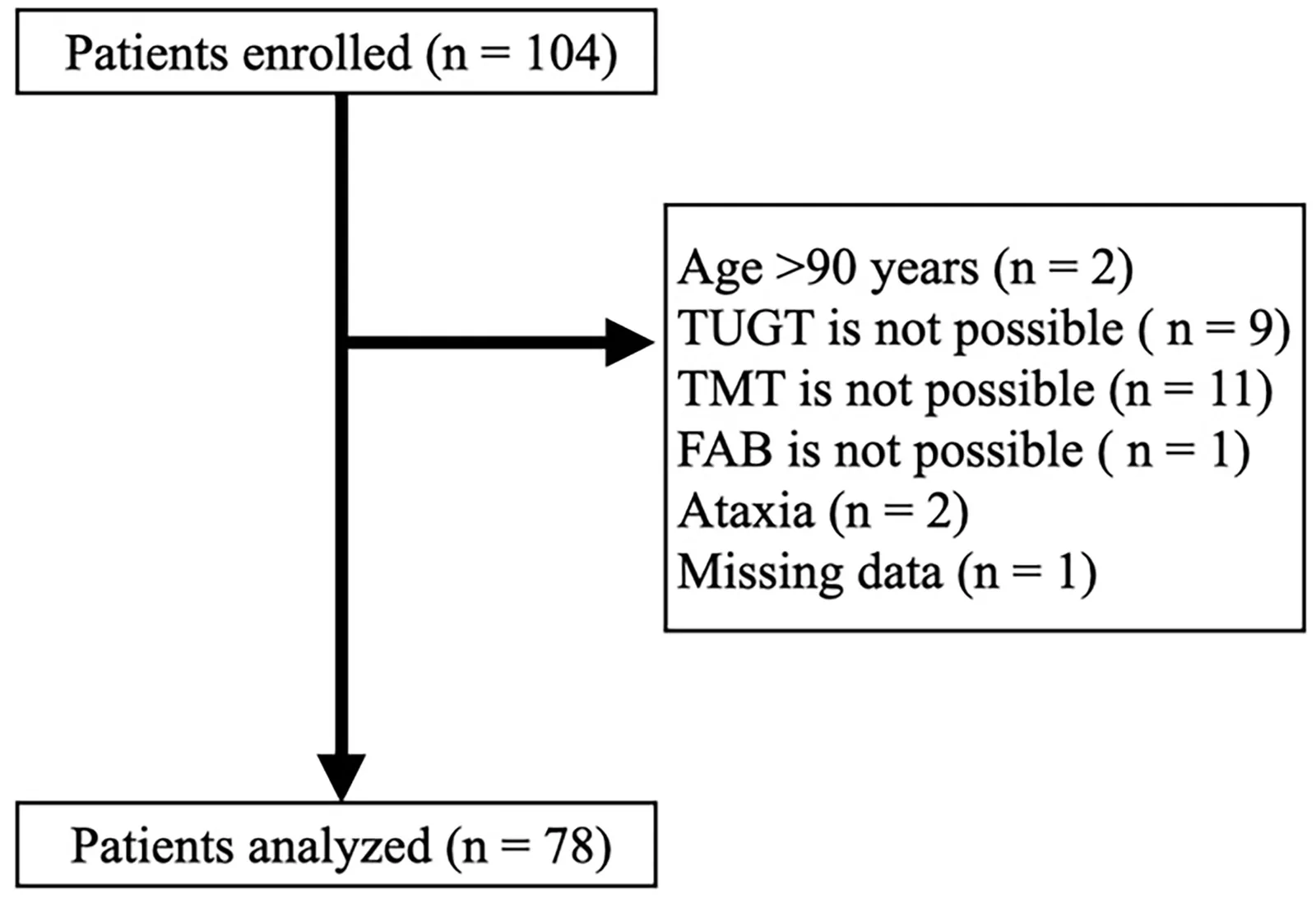
Patient flowchart. A total of 104 patients were enrolled, but 26 were excluded, leaving 78 for analysis.

The MDS stress value was 0.2, indicating that the model is within the acceptable range. The mean silhouette coefficients for k = 2, 3, 4, 5, and 6 were 0.526, 0.595, 0.586, 0.593, and 0.587, respectively (supplementary file 1). Based on the results of 1,000 bootstrap samples, 993 bootstrap samples yielded valid clustering solutions. The mean ARI was 0.405 ± 0.148, with a median of 0.401 and a 95% percentile interval of 0.144–0.702. The results of the k-means cluster analysis classified the data into three clusters (cluster 1: inhibitory control impairment group; cluster 2: motor programming impairment group and cluster 3: mild executive dysfunction group).

There were no significant differences in the basic characteristics among the three clusters (Fig. 3, Table 1). For the cognitive variables, the omnibus effect of cluster was not significant for FAB total score (F (2, 74) = 1.22, p = 0.302, partial η² = 0.032), MMSE (F (2, 74) = 0.07, p = 0.933, partial η² = 0.002), TMT part A (F (2, 74) = 0.11, p = 0.892, partial η² = 0.003), TMT part B (F (2, 64) = 1.81, p = 0.173, partial η² = 0.053), TMT part B−A (F (2, 64) = 1.65, p = 0.200, partial η² = 0.049), and FIM cognitive score (F (2, 74) = 0.05, p = 0.954, partial η² = 0.001). Pairwise adjusted mean differences and 95% confidence intervals are presented in Table 2.

**Fig. 3:**
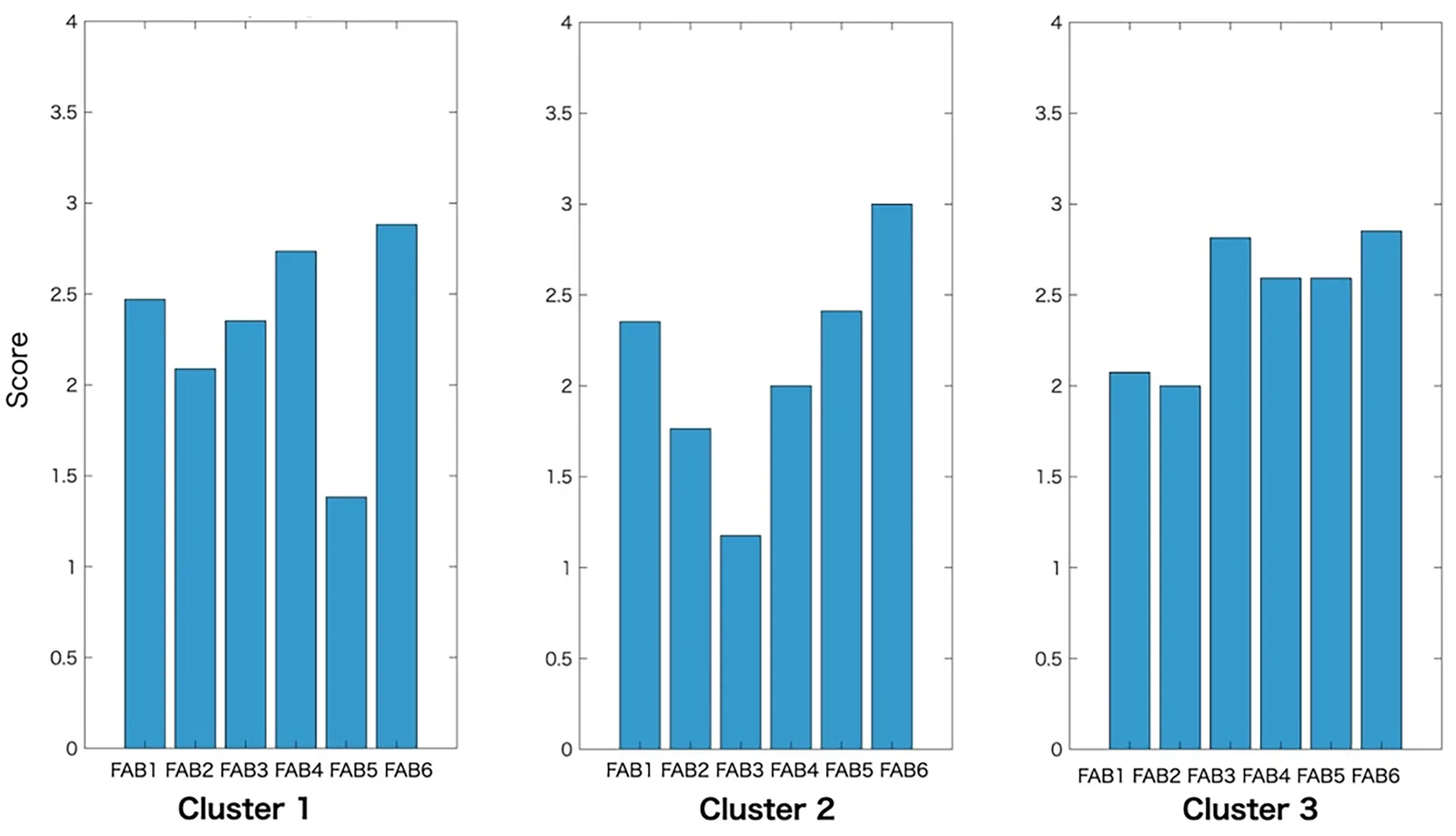
Structural similarities among the three groups. The vertical axis shows the FAB scores, and the horizontal axis shows the FAB items. In Cluster 1, FAB5 (inhibitory function) was relatively impaired; in Cluster 2, FAB3 (motor programming) was relatively impaired; and in Cluster 3, the impairment was mild.

**Table 1:** Basic attributes.

Variable	Total	Cluster 1	Cluster 2	Cluster 3	P value
	(N = 78)	(N = 32)	(N = 18)	(N = 28)	
Age (years)	70.3 ± 13.0	72.2 ± 12.3	71.5 ± 14.2	67.3 ± 12.9	-
Sex (male/female)	49/29	18/14	15/3	11/17	P = 0.143
Type (infarction/hemorrhage)	42/36	20/12	12/6	10/18	P = 0.335
Paralyzed side (right/left)	40/38	18/14	9/9	11/17	P = 0.420
Time since stroke (days)	55.4 ± 54.9	60.0 ± 36.8	61.0 ± 33.9	61.6 ± 72.4	P = 0.766

Data are expressed as mean ± standard deviation.

**Table 2:** Results of cognitive function assessments.

Variable	Total	Cluster 1	Cluster 2	Cluster 3	Between-cluster effect
	(N = 78)	(N = 32)	(N = 18)	(N = 28)	
FAB item 1 (score)	2.5 (0 - 3)	2 (1 - 3)	3 (1 - 3)	3 (1 - 3)	1 vs 2: OR 1.13 (0.35–3.62), p = 0.840 1 vs 3: OR 2.21 (0.80–6.06), p = 0.125 2 vs 3: OR 2.16 (0.67–6.96), p = 0.199
FAB item 2 (score)	2 (0 - 3)	3 (1 - 3)	2 (0 - 3)	2 (1 - 3)	1 vs 2: OR 0.68 (0.22–2.10), p = 0.508 1 vs 3: OR 1.54 (0.58–4.07), p = 0.388 2 vs 3: OR 1.22 (0.39–3.82), p = 0.727
FAB item 3 (score)	3 (0 - 3)	3 (1 - 3)	1 (0 - 3)	3 (0 - 3)	1 vs 2: OR 0.16 (0.05–0.54), p = 0.003 1 vs 3: OR 0.21 (0.06–0.75), p = 0.016 2 vs 3: OR 0.01 (0.001–0.09), p < 0.001
FAB item 4 (score)	3 (1 - 3)	3 (1 - 3)	2 (0 - 3)	3 (1 - 3)	1 vs 2: OR 0.34 (0.10–1.14), p = 0.082 1 vs 3: OR 3.39 (0.98–11.72), p = 0.054 2 vs 3: OR 0.13 (0.03–0.48), p = 0.002
FAB item 5 (score)	2 (0 - 3)	1 (0 - 3)	3 (1 - 3)	3 (1 - 3)	1 vs 2: OR 19.94 (5.04–78.91), p < 0.001 1 vs 3: OR 0.06 (0.02–0.20), p < 0.001 2 vs 3: OR 1.04 (0.28–3.81), p = 0.956
FAB item 6 (score)	3 (1 - 3)	3 (1 - 3)	3 (1 - 3)	3 (2 - 3)	1 vs 2: NE, p = NE 1 vs 3: NE, p = NE 2 vs 3: OR 1.34 (0.22–8.28), p = 0.755
FAB total (score)	13.7 ± 2.6	14.9 ± 2.9	13.2 ± 3.4	14.4 ± 2.7	partial η² = 0.032 1 vs 2: Δ = −1.08 (−2.59 to 0.43), p = 0.157 1 vs 3: Δ = −0.28 (−1.74 to 1.19), p = 0.708 2 vs 3: Δ = 0.81 (−0.50 to 2.11), p = 0.222
MMSE (score)	27.3 ± 3.2	27.5 ± 2.4	27.1 ± 2.6	27.7 ± 2.8	partial η² = 0.002 1 vs 2: Δ = −0.03 (−1.56 to 1.50), p = 0.971 1 vs 3: Δ = −0.23 (−1.71 to 1.24), p = 0.753 2 vs 3: Δ = −0.21 (−1.52 to 1.11), p = 0.756
TMT Part A (sec)	72.6 ± 37.4	65.9 ± 30.8	68.1 ± 32.9	62.5 ± 30.0	partial η² = 0.003 1 vs 2: Δ = −1.37 (−17.51 to 14.78), p = 0.866 1 vs 3: Δ = −3.57 (−19.20 to 12.05), p = 0.650 2 vs 3: Δ = −2.21 (−16.13 to 11.71), p = 0.753
TMT Part B (sec)	190.5 ± 184.2	132.7 ± 69.9	119.4 ± 69.2	126.9 ± 68.1	partial η² = 0.053 1 vs 2: Δ = −20.97 (−100.76 to 58.81), p = 0.604 1 vs 3: Δ = −67.78 (−146.21 to 10.65), p = 0.089 2 vs 3: Δ = −46.80 (−112.75 to 19.15), p = 0.161
TMT Part B - A (sec)	90.4 ± 118.7	124.3 ± 169.5	68.8 ± 54.4	62.9 ± 54.5	partial η² = 0.049 1 vs 2: Δ = −15.25 (−89.93 to 59.44), p = 0.684 1 vs 3: Δ = −59.11 (−132.53 to 14.32), p = 0.113 2 vs 3: Δ = −43.86 (−105.60 to 17.88), p = 0.161
FIM cognitive (score)	28.1 ± 6.8	29.6 ± 5.3	28.4 ± 6.7	29.4 ± 5.5	partial η² = 0.001 1 vs 2: Δ = −0.21 (−3.83 to 3.40), p = 0.907 1 vs 3: Δ = 0.26 (−3.24 to 3.76), p = 0.881 2 vs 3: Δ = 0.48 (−2.64 to 3.59), p = 0.762

Data are expressed as mean ± standard deviation or median (min–maximum). FAB, Frontal Assessment Battery; MMSE, Mini-Mental State Examination; TMT, Trail Making Test; FIM, Functional Independence Measure; Odds ratios, OR; NE, not estimable because of quasi-complete separation. For ordinal variables, ORs are from age-adjusted ordinal logistic regression. For continuous variables, adjusted mean differences (Δ) are from age-adjusted linear mixed-effects models. partial η² represents the omnibus main effect of cluster.

For the FAB items, age-adjusted ordinal logistic regression showed no significant differences between clusters for FAB items 1 and 2. For FAB item 3, significant differences were observed between Cluster 1 and Cluster 2 (OR = 0.16, 95% CI 0.05–0.54, p = 0.003), Cluster 1 and Cluster 3 (OR = 0.21, 95% CI 0.06–0.75, p = 0.016), and Cluster 2 and Cluster 3 (OR = 0.01, 95% CI 0.001–0.09, p < 0.001). For FAB item 4, a significant difference was observed between Cluster 2 and Cluster 3 (OR = 0.13, 95% CI 0.03–0.48, p = 0.002), whereas the differences between Cluster 1 and Cluster 2 and between Cluster 1 and Cluster 3 were not significant (P > 0.05). For FAB item 5, significant differences were observed between Cluster 1 and Cluster 2 (OR = 19.94, 95% CI 5.04–78.91, p < 0.001) and between Cluster 1 and Cluster 3 (OR = 0.06, 95% CI 0.02–0.20, p < 0.001), but not between Cluster 2 and Cluster 3 (OR = 1.04, 95% CI 0.28–3.81, p = 0.956). FAB item 6 could not be reliably estimated for the Cluster 1 comparisons because of quasi-complete separation.

Comparisons of motor and sensory functions across the three clusters are presented in Table 3. Age-adjusted ordinal logistic regression showed significant differences in BRS scores between Cluster 1 and Cluster 3 (upper limb: OR = 0.12, 95%CI 0.04–0.36, p < 0.001; hand: OR = 0.13, 95%CI 0.04–0.39, p < 0.001; lower limb: OR 0.10, 95% CI, 0.03–0.34, p < 0.001) and between Cluster 2 and Cluster 3 (upper limb: OR = 0.16, 95%CI 0.05–0.54, p = 0.003; hand: OR = 0.16, 95%CI 0.05–0.54, p = 0.003; lower limb: OR = 0.06, 95% CI, 0.02–0.27, p < 0.001) for the upper limb, hand, and lower limb. No significant differences were observed between Cluster 1 and Cluster 2 for any of these BRS (P > 0.05). For upper-limb kinesthetic sense, the OR was 0.20 (95%CI 0.06–0.69, p = 0.011) for Cluster 1 versus Cluster 2, and 0.15 (95% CI 0.04–0.57, p = 0.006) for Cluster 2 versus Cluster 3. For upper-limb position sense, the OR was 0.23 (95% CI, 0.06–0.90, p = 0.034) for Cluster 2 versus Cluster 3. For lower-limb kinesthetic sense, the OR was 0.26 (95% CI 0.08–0.92, p = 0.037) for Cluster 1 versus Cluster 3. For lower-limb position sense, the OR was 0.16 (95% CI 0.04–0.63, p = 0.009) for Cluster 1 versus Cluster 3. The remaining pairwise comparisons were not statistically significant (P > 0.05, Table 3).

**Table 3:** Results of physical function assessments.

Variable	Total	Cluster 1	Cluster 2	Cluster 3	Between-cluster effect
	(N = 78)	(N = 32)	(N = 18)	(N = 28)	
BRS Upper limb	5 (2 - 6)	5(2 - 6)	5 (2 - 6)	6 (3 - 6)	1 vs 2: OR 1.24 (0.43–3.54), p = 0.689 1 vs 3: OR 0.12 (0.04–0.36), p < 0.001 2 vs 3: OR 0.16 (0.05–0.54), p = 0.003
BRS Hand	5 (2 - 6)	5 (2 - 6)	5 (2 - 6)	6 (3 - 6)	1 vs 2: OR 1.46 (0.50–4.23), p = 0.491 1 vs 3: OR 0.13 (0.04–0.39), p < 0.001 2 vs 3: OR 0.15 (0.04–0.52), p = 0.003
BRS Lower limb	5 (2 - 6)	5 (2 - 6)	5 (3 - 6)	6 (2 - 6)	1 vs 2: OR 0.74 (0.25–2.18), p = 0.588 1 vs 3: OR 0.10 (0.03–0.34), p < 0.001 2 vs 3: OR 0.06 (0.02–0.27), p < 0.001
SIAS Kinesthetic sense (Upper)	3 (1 - 3)	3 (1 - 3)	2 (1 - 3)	3 (2 - 3)	1 vs 2: OR 0.20 (0.06–0.69), p = 0.011 1 vs 3: OR 0.69 (0.22–2.15), p = 0.524 2 vs 3: OR 0.15 (0.04–0.57), p = 0.006
SIAS Position sense (Upper)	3 (0 - 3)	3 (0 - 3)	3 (1 - 3)	3 (2 - 3)	1 vs 2: OR 0.99 (0.32–3.09), p = 0.993 1 vs 3: OR 0.22 (0.06–0.79), p = 0.021 2 vs 3: OR 0.23 (0.06–0.90), p = 0.034
SIAS Kinesthetic sense (Lower)	3 (0 - 3)	3 (0 - 3)	3 (1 - 3)	3 (1 - 3)	1 vs 2: OR 1.30 (0.39–4.36), p = 0.667 1 vs 3: OR 0.26 (0.08–0.92), p = 0.037 2 vs 3: OR 0.28 (0.06–1.19), p = 0.084
SIAS Position sense (Lower)	3 (0 - 3)	3 (0 - 3)	3 (1 - 3)	3 (1 - 3)	1 vs 2: OR 1.74 (0.52–5.83), p = 0.369 1 vs 3: OR 0.16 (0.04–0.63), p = 0.009 2 vs 3: OR 0.30 (0.07–1.33), p = 0.113
TUGT (Maximum speed)	16.8 ± 12.1	19.4 ± 13.0	20.8 ± 15.8	11.3 ± 4.1	partial η² = 0.108 1 vs 2: Δ =1.48 (−5.27 to 8.23), p = 0.664 1 vs 3: Δ = 9.10 (2.12 to 16.08), p = 0.011 2 vs 3: Δ = −7.62 (−13.63 to −1.61), p = 0.014
BBS	47.0 ± 7.6	43.8 ± 8.4	46.4 ± 7.2	51.1 ± 4.7	partial η² = 0.157 1 vs 2: Δ = −4.05 (−8.18 to 0.09), p=.055 1 vs 3: Δ =6.62 (3.05 to 10.18), p≤.001 2 vs 3: Δ = 2.57 (−1.43 to 6.57), p=.205
FIM motor	67.0 ± 15.1	61.6 ± 16.9	68.1 ± 14.3	72.4 ± 11.3	partial η² = 0.086 1 vs 2: Δ = −3.40 (−12.13 to 5.33), p = 0.440 1 vs 3: Δ = 9.78 (2.26 to 17.31), p = 0.012 2 vs 3: Δ = 6.38 (−2.07 to 14.83), p = 0.137

Data are expressed as mean ± standard deviation or median (min–maximum). BRS, Brunnstrom Stage; SIAS, Stroke Impairment Assessment Set; TUGT, Timed Up and Go Test; BBS, Berg Balance Scale; FIM, Functional Independence Measure; Odds ratios, OR. For ordinal variables, ORs are from age-adjusted ordinal logistic regression. For continuous variables, adjusted mean differences (Δ) are from age-adjusted linear mixed-effects models. Partial η² represents the omnibus main effect of cluster.

In the linear mixed-effects models, the main effect of cluster was significant for TUGT (F (2, 74) = 4.47, p = 0.015, partial η² = 0.108), BBS (F (2, 74) = 6.87, p = 0.002, partial η² = 0.157), and FIM motor score (F (2, 74) = 3.48, p = 0.036, partial η² = 0.086), but not for FIM cognitive score (P > 0.05). For TUGT, significant differences were observed between Cluster 1 and Cluster 3 (adjusted mean difference = 7.62 s, 95% CI 1.61 to 13.64, p = 0.014) and between Cluster 2 and Cluster 3 (adjusted mean difference = 9.10 s, 95% CI 2.12 to 16.08, p = 0.011). The difference between Cluster 1 and Cluster 2 was not significant (adjusted mean difference = −1.48 s, 95% CI −8.23 to 5.28, p = 0.664). For BBS, the adjusted mean difference between Cluster 1 and Cluster 3 was 6.62 (3.05 to 10.18), p < 0.001). For FIM motor score, the adjusted mean difference between Cluster 1 and Cluster 3 was 9.78 (95% CI 2.26–17.31, p = 0.012).

Residual diagnostic analyses indicated deviations from normality for TMT part B, TMT B−A, and TUGT. The Q–Q plots showed departures from the reference line, particularly in the tails of the residual distributions. The Shapiro–Wilk tests were significant for TMT part B (W = 0.692, p < 0.001), TMT B−A (W = 0.646, p < 0.001), and TUGT (W = 0.789, p < 0.001). Residual-versus-fitted value plots indicated several observations with relatively large residuals and some variation in residual dispersion, particularly for these three outcomes. However, the Breusch–Pagan tests did not indicate statistically significant heteroscedasticity for TMT part B (LM = 3.44, p = 0.329), TMT B−A (LM = 3.30, p = 0.347), and TUGT (LM = 5.30, p = 0.151, supplementary file 2).

Sensitivity analyses for TMT part B and TMT B−A yielded results consistent with the primary analyses. For TMT part B, the cluster effect remained non-significant in the sensitivity analysis (F (2,64) = 1.27, p = 0.287) and the HC3 analysis (F (2,64) = 1.87, p = 0.162). Similarly, for TMT B−A, the cluster effect remained non-significant in the sensitivity analysis (F (2,64) = 1.04, p = 0.358) and the HC3 analysis (F (2,64) = 1.63, p = 0.204). For TUGT, the cluster effect remained significant in the robust analysis using HC3 standard errors (F (2,74) = 8.025, p < 0.001, supplementary file 2). The direction of the pairwise differences was consistent across the analyses, with TUGT values being higher in Cluster 1 and Cluster 2 than in Cluster 3. In the robust analysis, the corresponding differences on the original scale remained significant (Cluster 1 vs. Cluster 3: 95% CI, 3.09 to 12.15, p = 0.001; Cluster 2 vs. Cluster 3: 95% CI, 1.43 to 16.77, p = 0.020).

## Discussion

This study employed the FAB subscales to investigate, via RSA, whether specific patterns exist in score distributions and how these patterns relate to cognitive and physical functions in patients with stroke. RSA and cluster analysis identified three distinct structural subgroups. The distribution of individual FAB subitems varied significantly between clusters. This heterogeneity was specifically associated with differences in physical function in patients with stroke. Based on these characteristics, the clusters were defined as follows: Cluster 1, the “Inhibitory Control Impairment Group”; Cluster 2, the “Motor Programming Impairment Group”; and Cluster 3, the “Mild Executive Dysfunction Group”. A key finding was that despite equivalent total FAB scores, Cluster 1 exhibited relatively lower performance on FAB item 5 (Go/No-go), whereas Cluster 2 showed relatively lower performance on FAB item 3 (Luria’s motor series), indicating distinct patterns of FAB subitem performance. However, these subitems should not be regarded as direct or pure measures of isolated executive functions. Although FAB item 5 is commonly interpreted as reflecting response inhibition and. FAB item 3 involves motor sequencing and programming, performance on these tasks may also depend on multiple interacting cognitive and motor processes. Therefore, the present findings should be interpreted as differences in clinical performance profiles rather than definitive evidence of isolated inhibitory or motor-programming dysfunction. Furthermore, Cluster 3 demonstrated significantly superior performance in motor and sensory functions, balance, and ADL compared with Clusters 1 and 2, which both exhibited lower physical function. Importantly, MMSE, TMT part A, TMT part B, and FIM-cognitive scores did not significantly differ among the clusters. Thus, the differences in physical function and FAB subitem distributions were not accompanied by differences in these conventional global or composite cognitive measures. This finding suggests that examining the distribution of individual FAB subitem scores may reveal clinically relevant heterogeneity that is not readily captured by overall cognitive scores alone. Rather than indicating greater overall cognitive impairment in one cluster, the present findings suggest that patients with similar levels of global cognitive performance may exhibit different configurations of performance across specific FAB subitems, which may be associated with different levels of physical function. Taken together, these findings suggest that structural variations in FAB score distributions are associated with physical function, even in the absence of differences in overall executive function scores. This study results demonstrate that clinical evaluation should not rely solely on the FAB total score; instead, analyzing the distribution of subitem scores clarifies which specific components of executive function are associated with physical function. This approach provides a more nuanced interpretation of clinical conditions and can better inform the selection of targeted rehabilitation strategies. Traditional linear comparisons often fail to capture these nuances because individual strengths and weaknesses in sub-components—such as motor programming versus inhibitory control—cancel each other out in the total score. By employing RSA, we demonstrated that the structural distribution of executive components provides more granular information than composite scores. This highlights the clinical necessity of a "fractionated" approach to executive assessment, as proposed in contemporary multidimensional models of the frontal lobe.

The emergence of Cluster 1 ("Inhibitory Control Impairment Group ") and Cluster 2 ("Motor Programming Impairment Group ") may provide insight into the different clinical characteristics underlying functional deficits. Cluster 1 showed relatively lower performance on FAB item 5 (Go/No-go). The Go/No-go task is commonly used as a clinical indicator of response inhibition because it requires participants to suppress a prepotent response when prompted not to respond (Dubois et al., 2000). Motor and balance impairments associated with inhibitory deficits are common in patients with stroke. Yu et al (2021). evaluated inhibitory deficits in patients with stroke using the Stroop Color-Word Test to examine their association with TUGT performance and walking ability. Their findings demonstrated that inhibitory deficits were significantly associated with both TUGT performance and balance function. Furthermore, intervention studies support the hypothesis that inhibitory deficits underlie motor impairments. Yu et al (2022). demonstrated that repetitive transcranial magnetic stimulation applied to the dorsolateral prefrontal cortex resulted in concomitant improvements in both inhibitory control and motor function. These findings provide strong evidence for a functional link between inhibitory deficits and motor impairments. This suggests that motor impairment is not defined solely by muscle weakness or paralysis, but may also involve a decline in the ability to suppress unnecessary motor output and compensatory responses, as well as to select and switch between appropriate movement patterns. In current study, while motor paralysis (BRS) in this group was similar to Cluster 2, the qualitative nature of their functional impairment may differ, potentially involving impulsivity or difficulty in suppressing inappropriate motor responses during complex tasks like the TUGT. Nevertheless, the present study was cross-sectional and did not directly measure inhibitory control independently of other cognitive and motor processes. Therefore, the association observed between the Cluster 1 FAB profile and physical function should not be interpreted as evidence that impaired inhibitory control causes motor impairment. Rather, the findings indicate that a particular pattern of performance on the Go/No-go subitem co-occurred with lower physical function.

Cluster 2 was characterized by relatively lower performance on FAB item 3. This task is based on Luria's sequence, and requires learning the sequence as well as the temporal organization, maintenance, and execution of a series of movements (Dubois et al., 2000). There are various sequence tasks, but in each one, participants need to learn the sequence and perform the task (Prasomsri et al., 2024; Dahms et al., 2020). For example, motor learning was associated with motor deficits in patients with stroke. When Russo et al (2021). administered a sequential finger-tapping task to patients with stroke, they found that the level of motor impairment correlated with the extent of learning. In addition, it has been reported that executive dysfunction is associated with motor paralysis and balance function in patients with stroke (Friedman, & Miyake, 2017; Hayes et al., 2016). The significant association between this cluster and poorer motor recovery (BRS) and balance (BBS) suggests that a deficit in organizing motor sequences associated with impaired postural control and motor execution. Additionally, this cluster had sensory impairments as well as motor programming deficits. Sensory feedback provides information about the actual state of the limbs and is crucial for comparing predicted and observed movement states during the processes of motor learning and control (Kwon et al., 2019; Moore et al., 2024). Therefore, impairments in proprioception may reduce the accuracy of this sensorimotor comparison and hinder the acquisition of motor sequences. However, it is not possible to determine whether the low scores on FAB item 3 contribute to impairments in motor function and balance, whether impairments in motor function affect performance on the task, or whether both are influenced by other common factors. Therefore, the observed association should be interpreted as a coexisting clinical pattern rather than a causal relationship.

Furthermore, although this study found differences in the distribution of individual FAB subscales and in physical function, no significant differences were observed between clusters in the MMSE, TMT part A and B, or FIM cognitive scores. This suggests that conducting profile-based analyses of individual FAB subscales may reveal clinically significant heterogeneity that cannot be fully captured by overall or composite cognitive function measures. Therefore, the value of this approach lies not in identifying differences in overall cognitive impairment, but in characterizing distinct patterns in the performance of cognitive tasks that may be associated with differences in physical function.

Several limitations warrant consideration. First, this study did not incorporate neuroimaging analysis, such as voxel-based lesion-symptom mapping. Identifying the exact lesion locations associated with each cluster would strengthen the neuroanatomical claims. Second, the cross-sectional design prevents us from determining how these executive profiles evolve during the chronic phase. Future longitudinal studies are needed to examine whether improving specific executive components leads to better motor outcomes. Third, this study was conducted at three facilities and involved a population with relatively high levels of physical function. Therefore, its generalizability is limited. Forth, although the three-cluster solution had the highest mean silhouette coefficient among the evaluated solutions (k = 2–6), the bootstrap analysis yielded a mean ARI of 0.405, indicating variability in cluster membership across resampled datasets. Fifth, the identified clusters should be interpreted as exploratory phenotypic patterns rather than definitive and highly stable subgroups. The relatively small sample size and unequal cluster sizes may have contributed to the observed variability in cluster membership. Future studies with larger samples and independent cohorts are needed to examine the reproducibility and generalizability of these phenotypic clusters. Finally, although age was included as a covariate, this study did not account for several potentially important confounding factors. These include stroke severity, lesion location, the extent of stroke-induced brain volume loss, the intensity of rehabilitation, educational level, and pre-onset cognitive status. While stroke severity and lesion characteristics may affect both executive and motor functions, educational level and pre-onset cognitive status may influence performance on cognitive assessments. Consequently, residual confounding may contribute to the differences in clinical outcomes observed between clusters. Future studies should incorporate standardized measures of stroke severity, lesion characteristics, rehabilitation status, educational background, and pre-onset cognitive function in larger longitudinal cohorts.

## Conclusion

The distribution of FAB sub-item scores reveals distinct executive profiles that are closely associated with motor and balance impairments in patients with stroke. Relying solely on the FAB total score may overlook these critical differences. In order to gain a comprehensive understanding of a patient’s clinical condition and develop targeted, effective rehabilitation strategies, it is necessary to conduct a more detailed assessment of each component of executive function.

## Funding

Not applicable.

## Data availability statement

Data available on request from the authors.

## Declaration of competing interest

The authors declare that they have no known competing financial interests or personal relationships that could have appeared to influence the work reported in this paper.

## References

[bib0001] Berg K., Wood-Dauphinee S., Williams J.I. (1995). The balance scale: Reliability assessment with elderly residents and patients with an acute stroke. Scandinavian Journal of Rehabilitation Medicine.

[bib0002] Brunnstrom S. (1966). Motor testing procedures in hemiplegia: Based on sequential recovery stages. Physical Therapy.

[bib0003] Divandari N., Bird M-L, Jaberzadeh S. (2023). The association between cognitive domains and postural balance among healthy older adults: A systematic review of literature and meta-analysis. Current Neurology and Neuroscience Reports.

[bib0004] Dahms C., Brodoehl S., Witte O.W., Klingner CM. (2020). The importance of different learning stages for motor sequence learning after stroke. Human Brain Mapping.

[bib0005] Dubois B., Slachevsky A., Litvan I., Pillon B. (2000). The FAB: a frontal assessment battery at bedside. Neurology.

[bib0006] Friedman N.P., Miyake A. (2017). Unity and diversity of executive functions: Individual differences as a window on cognitive structure. Cortex; A Journal Devoted to the Study of the Nervous System and Behavior.

[bib0007] Granger C. (1986). Guide for use of uniform data set for medical rehabilitation. Buffalo General Hospital.

[bib0008] Han M., Kim D.Y., Leigh J.H., Kim MW. (2020). Value of the frontal assessment battery tool for assessing the frontal lobe function in stroke patients. Annals of Rehabilitation Medicine.

[bib0009] Hafsteinsdottir T., Rensink M., Schuurmans M. (2014). Clinimetric properties of the timed up and go test for patients with stroke: a systematic review. Topics in Stroke Rehabilitation.

[bib0010] Hayes S., Donnellan C., Stokes E. (2011). The measurement and impairment of executive function after stroke and concepts for physiotherapy. Physical Therapy Reviews : PTR.

[bib0011] Hayes S., Donnellan C., Stokes E. (2013). Associations between executive function and physical function poststroke: A pilot study. Physiotherapy.

[bib0012] Hayes S., Donnellan C., Stokes E. (2016). Executive dysfunction and balance function post-stroke: A cross-sectional study. Physiotherapy.

[bib0013] Kodinariya T., Makwana P. (2013). Review on determining of cluster in K-means clustering. Int J Adv Res Computer Science and Management Studies.

[bib0014] Kwon Y., Shin J.Y., Son SM. (2019). Deficit of motor skill acquisition on the upper limb ipsilesional to the injured hemisphere in individuals with stroke. Medical Science Monitor : International Medical Journal of Experimental and Clinical Research.

[bib0015] Lipskaya-Velikovsky L., Zeilig G., Weingarden H., Rozental-Iluz C., Rand D. (2018). Executive functioning and daily living of individuals with chronic stroke: measurement and implications. International Journal of Rehabilitation Research. Internationale Zeitschrift fur Rehabilitationsforschung. Revue Internationale de Recherches de Readaptation.

[bib0016] Liu M., Chino N., Tuji T., Masakado Y., Hase K., Kimura A. (2002). Psychometric properties of the stroke impairment assessment set (SIAS). Neurorehabilitation and Neural Repair.

[bib0017] Miyake A., Friedman N. (2012). The nature and organization of individual differences in executive functions: four general conclusions. Current Directions in Psychological Science.

[bib0018] Moore R.T., Piitz M.A., Singh N., Dukelow S.P., Cluff T. (2024). The independence of impairments in proprioception and visuomotor adaptation after stroke. Journal of Neuroengineering and Rehabilitation.

[bib0019] Nili H., Wingfield C., Walther A., Su L., Marslen-Wilson W., Kriegeskorte N. (2014). A toolbox for representational similarity analysis. PLoS Computational Biology.

[bib0020] Sakai K., Hosoi Y., Harada Y. (2023). Walking ability associated with executive dysfunction in patients with stroke: a cross-sectional study. Brain Sciences.

[bib0021] Sakai K., Hosoi Y., Harada Y., Kato Y. (2024). Association between the executive dysfunction and balance function in patients with stroke. Brain and Behavior.

[bib0022] Sakai K., Hosoi Y., Harada Y., Kato Y., Miyauchi T. (2025). Components of frontal assessment battery and clinical features in patients with stroke. Journal of Rehabilitation Medicine.

[bib0023] Sakai K., Hosoi Y., Harada Y., Morikawa K., Kato Y. (2024). Validity and reliability of the Japanese version of the frontal assessment battery in patients with stroke. Neurology International.

[bib0024] Sakai K., Hosoi Y., Harada Y., Ikeda Y. (2023). Estimation error consisting of motor imagery and motor execution in patients with stroke. Journal of Motor Behavior.

[bib0025] Sakai K., Hosoi Y., Harada Y., Ikeda Y., Tanabe J. (2024). Overestimation associated with walking and balance function in individuals diagnosed with a stroke. Physiotherapy Theory and Practice.

[bib0026] Sakai K., Hosoi Y., Tanabe J. (2024). Intervention and assessment of executive dysfunction in patients with stroke: a scoping review. PloS One.

[bib0027] Sakai K., Kawasaki T., Kim K., Ogawa J., Yamaguchi Y. (2026). Subtype of the Freezing of gait and motor function in patients with Parkinson's Disease. Movement Disorders Clinical Practice.

[bib0028] Skidmore E.R., Eskes G., Brodtmann A. (2023). Executive function poststroke: Concepts, recovery, and interventions. Stroke.

[bib0029] Tamez E., Myerson J., Morris L., White D.A., Baum C., Connor LT. (2011). Assessing executive abilities following acute stroke with the trail making test and digit span. Behavioural Neurology.

[bib0030] Ownsworth T., Shum D. (2008). Relationship between executive functions and productivity outcomes following stroke. Disability and Rehabilitation.

[bib0031] Pacheco-Estefan D., Fellner M.C., Kunz L., Zhang H., Reinacher P., Roy C., Brandt A., Schulze-Bonhage A., Yang L., Wang S., Liu J., Xue G., Axmacher N. (2024). Maintenance and transformation of representational formats during working memory prioritization. Nature Communications.

[bib0032] Perneczky R., Wagenpfeil S., Komossa K., Grimmer T., Diehl J., Kurz A. (2006). Mapping scores onto stages: Mini-Mental State Examination and clinical dementia rating. The American journal of geriatric psychiatry : official journal of the American Association for Geriatric Psychiatry.

[bib0033] Prasomsri J., Sakai K., Ikeda Y., Keereena S. (2024). Effectiveness of implicit learning in stroke rehabilitation: a systematic review. Physical Therapy Reviews : PTR.

[bib0034] Russo C., Veronelli L., Casati C., Monti A., Perucca L., Ferraro F., Corbo M., Vallar G., Bolognini N. (2021). Explicit motor sequence learning after stroke: A neuropsychological study. Experimental Brain Research.

[bib0035] Ward JH. (1963). Hierarchical grouping to optimize an objective function. Journal of the American Statistical Association.

[bib0036] Wang T-L, Hung Y-H, Yang C-C. (2016). Psychometric properties of the Taiwanese (Traditional Chinese) version of the frontal assessment battery: A preliminary study. Applied neuropsychology.

[bib0037] Yu H., Liu S., Dai P., Wang Z., Lan Y., Liu C., Zhang H. (2022). Effects of repetitive transcranial magnetic stimulation on gait and postural control ability of patients with executive dysfunction after stroke. Brain Sciences.

[bib0038] Yu H., Zhang Q., Liu S., Dai P., Lan Y., Xu G., Zhang H. (2021). Effect of executive dysfunction on posture control and gait after stroke. Evidence-Based Complementary and Alternative Medicine : eCAM.

[bib0039] Yuan C., Yang H. (2019). Research on k-value selection method of k-means clustering algorithm. J.

