# Supplementary material for: Physical function varies according to characteristics of executive dysfunction in patients with stroke
Source: Int J Clin Health Psychol. 2026 Sep 26;26(3):100725. doi: 10.1016/j.ijchp.2026.100725 (PMC13635640; doi:10.1016/j.ijchp.2026.100725)
Supplement: Supplementary file 1 [file mmc1.docx]

Supplementary file 1

The mean silhouette coefficients for k = 2, 3, 4, 5, and 6 were 0.526, 0.595, 0.586, 0.593, and 0.587, respectively.
