# Supplementary material for: Physical function varies according to characteristics of executive dysfunction in patients with stroke
Source: Int J Clin Health Psychol. 2026 Sep 26;26(3):100725. doi: 10.1016/j.ijchp.2026.100725 (PMC13635640; doi:10.1016/j.ijchp.2026.100725)
Supplement: Supplementary file 2 [file mmc2.docx]

Supplementary file 2

| Table S1-1. Diagnostic assessment of the linear mixed-effects models | | | | | | | | | |
| --- | --- | --- | --- | --- | --- | --- | --- | --- | --- |
| Outcome | Shapiro–Wilk W | Shapiro–Wilk p-value | Breusch–Pagan | | Breusch–Pagan p-value | Maximum absolute standardized residual | | Maximum Cook's distance | |
| TMT part B | 0.692 | < 0.001 | | 3.44 | 0.329 | | 5.99 | | 0.376 |
| TMT B−A | 0.646 | < 0.001 | | 3.3 | 0.347 | | 6.18 | | 0.4 |
| TUGT | 0.789 | < 0.001 | | 5.3 | 0.151 | | 4.37 | | 0.278 |

TMT: Trail Making Test, TUGT: Timed Up and Go Test.

| Table S1-2. Sensitivity analyses of continuous outcomes showing deviations from model assumptions | | | | | | |
| --- | --- | --- | --- | --- | --- | --- |
| Outcome | Primary LMM: F | Primary LMM: p-value | Sensitivity analysis: F | Sensitivity analysis: p-value | Robust HC3: F | Robust HC3: p-value |
| TMT part B | 1.805 | 0.173 | 1.273 | 0.287 | 1.873 | 0.162 |
| TMT B−A | 1.652 | 0.200 | 1.043 | 0.358 | 1.627 | 0.204 |
| TUGT | 4.474 | 0.015 | 6.156 | 0.003 | 8.025 | < 0.001 |

LMM: linear mixed-effects models, HC: heteroscedasticity-consistent. TMT: Trail Making Test, TUGT: Timed Up and Go Test.
